# Extraction and Application of Hydraulic Support Safety Valve Characteristic Parameters Based on Roof Pressure Data

**DOI:** 10.3390/s23218853

**Published:** 2023-10-31

**Authors:** Keke Xing, Jingyi Cheng, Zhijun Wan, Xin Sun, Wanzi Yan, Jiakun Lv, Minti Xue

**Affiliations:** 1 School of Mines, China University of Mining and Technology, Xuzhou 221116, China; tb20020032b4@cumt.edu.cn (K.X.); zhijwan@cumt.edu.cn (Z.W.); tb21020042b0@cumt.edu.cn (X.S.); wzyan@cumt.edu.cn (W.Y.); jiakun_l11b4@cumt.edu.cn (J.L.); ts22020089a31@cumt.edu.cn (M.X.); 2 Artificial Intelligence Research Institude, China University of Mining and Technology, Xuzhou 221116, China

**Keywords:** safety valve, roof pressure data, state monitoring, amplitude threshold method

## Abstract

The safety valves of powered supports control the maximum working resistance, and their statuses must be known to ensure the safety of both the support and the overlying strata. However, the inspection of powered support valves involves manual or semiautomated operations, the costs of which are high. In this study, an extreme point extraction method was developed for the determination of the characteristic parameters of safety valves using roof pressure data, and a safety valve state monitoring module was constructed. Using the longwall face of 0116^3^06 with top coal caving in the Mindong Mine as an example, the characteristic parameters of the safety valves were extracted, including the peak, reseating, and blowdown pressures, as well as the recovery and unloading durations. The results of the field tests showed the following: (1) The amplitude threshold method based on extreme points can be used to accurately extract characteristic parameters, and the distribution of the characteristic parameters of the safety valves follows either a Gaussian or an exponential distribution. (2) The mining pressure analysis results, derived from the characteristic parameters, closely align with the in situ mining pressure observations. This method can be used for the online monitoring of safety valve conditions, increasing the operational efficiency and quality of safety valve inspections.

## 1. Introduction

The powered supports in a longwall mining system must be compatible with the requirements to control the roof and floor to ensure the safety of the machines and workers [1]. Powered roof supports are divided into units, which consist of mechanical structural components, hydraulic systems, and electrohydraulic control systems. The main element of the hydraulic system of powered supports is the hydraulic legs, which extend between the canopy and sill piece [2,3,4]. These hydraulic legs ensure that an adequate force is maintained on the roof of the longwall face in the section. Protection against excessive pressure is provided by a special safety valve. This safety valve governs the maximum working resistance of the system; its adaptability and reliability are critical factors that influence the safety of both the support structure and roof [5].

Although safety valves undergo random inspections before delivery, situations arise in which the safety valve fails to align with the roof strata movement, and faults such as safety valve nonactuation and leakage can occur, leading to the degradation of the support–roof strata relationship. Therefore, a study on the monitoring of the state of powered support safety valves was conducted utilizing high-frequency and high-precision roof pressure data.

Currently, there is a comprehensive range of support safety valves that have been domestically developed, which satisfactorily fulfill the diverse flow rate requirements of mining operations. For the case in which panels experience substantial mining pressure, various forms of large-capacity safety valves have been designed and developed, including direct action, differential action, and pilot action, where the nominal flow rate can reach up to 2000 L/min [6]. In terms of the condition monitoring and fault diagnosis of safety valves, Lu [7] employed a wavelet method to extract and analyze critical points from the time–pressure curve of the safety valve testing system. Liu, R.Y. [8] proposed a noise-robust algorithm to analyze the transient flow from permanent downhole pressure data. Zhang, W. [9] used acoustic emission technology for the online monitoring and fault diagnosis of safety valve leakages. Louzada Francisco and Danilo Colombo et al. [10,11] developed the FEMaR regression model to analyze the faults and reliability of safety valves in oil wells. Cheng, J.Y. [12,13] emphasized the significance of the opening characteristics of safety valves to ensure powered support safety. Xu, G. [14] developed a powered support state evaluation model that incorporates the safety valve’s opening rate. Liu Z [15] analyzed safety valve nonactuation faults using roof pressure data. Szurgacz, D. [16,17] analyzed the pressure increase and internal pressure leaks in the hydraulic cylinder of a longwall powered roof support during use and determined the operating range of the safety valve through bench tests conducted on the prop and the safety valve under dynamic loads.

The characteristic parameters of safety valves mainly include both static characteristics, such as the opening pressure, reseating pressure, and blowdown pressure, and dynamic characteristics, such as the impact peak pressure, recovery time, unloading time, and pressure overshoot [18]. During the practical use of powered support safety valves, static performance indicators are employed to determine the precision of valve opening and closing and deviations in the setting force. Dynamic performance indicators were employed to assess the safety valve’s sensitivity and shock resistance. Furthermore, the frequency of safety valve actuations was quantified to assess the roof weighting strength and the distribution patterns of the inclined panel pressure. Moreover, the status of the safety valve was statistically analyzed while considering the evolutions of the characteristics in conjunction with the frequency of valve opening.

The following issues with underground support safety valves in coal mines remain to be addressed: (1) The on-site assessments of safety valve overflow primarily rely on manual inspections, serving as a specific indicator of the roof pressure. (2) Routine assessments of the safety valve conditions and pressure calibration are required, resulting in elevated maintenance costs. (3) The absence of foundational data for safety valves under actual operational conditions hinders precise evaluations of their compatibility with roof movement.

In this study, taking the direct-acting relief valve as an example, which is a commonly used component in powered support systems, both the dynamic and static characteristics of this safety valve were analyzed. From the results, the principles and techniques needed to extract its characteristic parameters based on the roof pressure data were determined. Furthermore, the relationship between the characteristic parameters and strata pressure was clarified. Subsequently, the safety valve’s status was analyzed and assessed. Finally, a safety valve status monitoring module was developed to enable the real-time monitoring of the status of safety valves.

## 2. Safety Valve Characteristic Parameter Extraction Principles

### 2.1. Structure and Mathematical Model of Safety Valve

**Figure 1:**
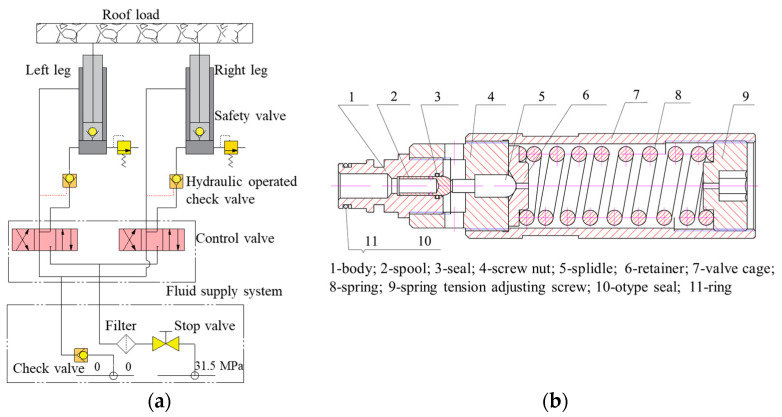
Schematic diagram of hydraulic system and structural model of safety valve: (**a**) hydraulic system for support columns; (**b**) simplified structural diagram of fad320/40 spring-loaded safety valve.

The hydraulic system of the support column is illustrated in Figure 1a. This system primarily comprises components including a fluid supply system, control valve, column, hydraulic-operated check valve, safety valve, and check valve [5]. Safety valves with high precision and reliability are required to discharge and maintain pressure under conditions where the roof may descend slowly or rapidly, thereby guaranteeing the constant-resistance of the support. Commonly used spring-loaded safety valves are shown in Figure 1b, which feature a quick-plug connection and an internal structure based on direct-acting spring principles. The flow rate of the safety valve is adjusted using an outlet aperture and the number of outlet ports in the interaction between the emulsion pressure upstream of the valve port and the force exerted by the spring (8) on the spool (2) [19]. In this study, the FAD320/40 safety valve was the subject, which has a nominal flow rate of 320 L/min and a nominal pressure of 40 MPa.

In the process of panel mining, the key upper rock strata undergo periodic fracturing and instability, resulting in roof collapse and a marked rise in powered support resistance. When the roof structure experiences extensive instability, swift subsidence of the immediate roof may occur, with notable alterations in the leg pressure and volume. Consequently, the safety valve continuously and rapidly discharges over a broad range. Its primary purpose is to uphold the leg pressure at the preset level. However, variations in the input flow rate to the safety valve result in changes in valve opening and the compression of the pressure-regulating spring. Consequently, both the hydraulic and spring forces fluctuate. Additionally, during the operation of the safety valve, fluctuations in the overflow rate lead to pressure variations within the system. The accuracy of a valve’s pressure regulation is determined through analyzing the pressure–flow characteristics of the safety valve. Based on the principles of dynamics and continuity, a mathematical model for the pressure–flow characteristics of the safety valve was established as follows:

The continuity equation for the safety valve is

(1) $$Q_{1}-k_{L}P=C_{1}\frac{dP}{dt}+A_{0}\frac{dX}{dt}+Q$$

where $Q_{1}$ is the inflow rate to the system, $k_{L}$ is the system’s leakage coefficient, $C_{1}$ is the fluid capacity of the hydraulic pipeline and valve chamber, $A_{0}$ is the valve core’s pressure-bearing surface area, and X is the valve core displacement.

The flow equation for the overflow port is

(2) $$Q=C_{d}\pi dX\sqrt{2P/\rho }$$

where Q is the overflow rate, $C_{d}$ is the flow coefficient, d is the valve core diameter, X is the valve port span, P the is valve chamber pressure, and ρ is the emulsion density.

The dynamic force equilibrium equation of the safety valve spool is

(3) $$PA_{0}-F_{y}-K(X+X_{0})=m\frac{d^{2}X}{dt^{2}}+R_{fa}\frac{dX}{dt}$$

where $X_{0}$ is the precompression of the spring, m is the mass of the valve core, $R_{fa}$ is the equivalent valve core damping coefficient, $F_{y}=K_{dynamic}\times X$ is the hydraulic force, and K is the spring stiffness.

### 2.2. Simulation Analysis of the Dynamic–Static Characteristics of Safety Valve

#### 2.2.1. Establishment of AMESim Simulation Model

AMESim is commonly used to simulate the supports of hydraulic systems [20]. This study selected the ZF4800/17/28 top coal caving support column as the object. The liquid supply system employed a water-in-oil emulsion with a 5% emulsion content. The pump station was rated at a supply pressure of 31.5 MPa and a flow rate of 400 L/min. The column consisted of a single telescopic cylinder with a cylinder diameter/column diameter of 200/185 mm. The hydraulic check valve was an FDYM200/50, with a rated flow rate of 200 L/min. The control valve was a three-position, four-way reversing valve with a 2 mm positive overlap. The safety valve was an FAD320/40 featuring an 8 mm spool diameter, a maximum spool displacement of 8 mm, eight 2 mm liquid ports around the spool, a 6 mm zero-displacement positive overlap, and a spring stiffness of 1144 N/mm.

**Figure 2:**
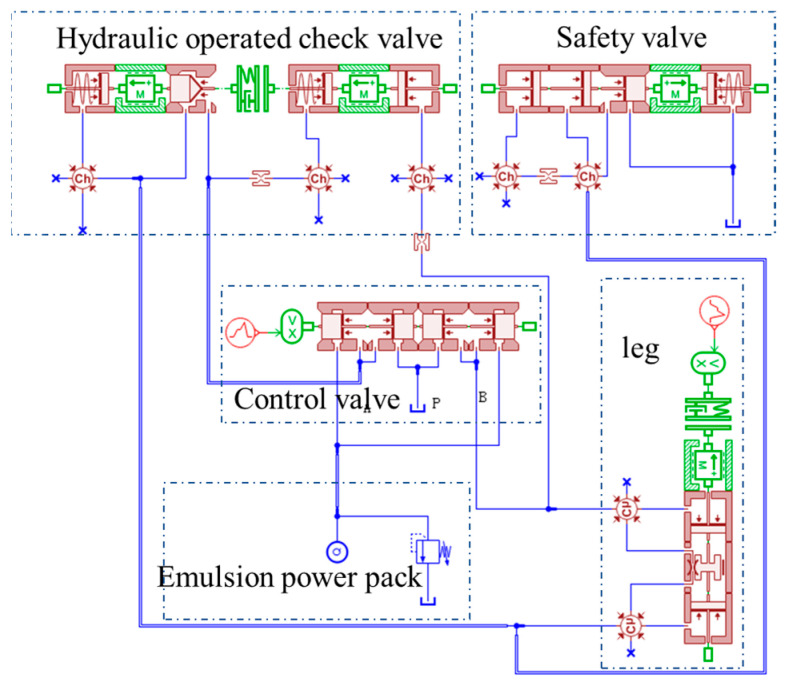
AMESim simulation model: powered support leg (with safety valve).

Figure 2 illustrates the simulation model of the ZF4800/17/28 top coal caving powered support column, with the specific model parameters provided in Table 1. The powered support simulation model comprises the emulsion power pack; the three-position, four-way control valve; column; safety valve; hydraulic operated check valve; and associated pipelines. Due to the intricate field conditions, the simulation was conducted under the following assumptions: (1) the viscosity and density of the emulsion remained constant; (2) the influence of air pockets was disregarded; and (3) the safety valve’s outlet was directly linked to the oil tank, maintaining an outlet pressure equivalent to the atmospheric pressure.

#### 2.2.2. Analysis of Static Characteristics of Safety Valves

The primary parameters used to assess the static performances of the safety valves are the pressure–flow characteristics, which are attributes related to the opening, overflow, and closure between the low and nominal flows. According to the MT419-1995 standard [21], the pressure under low-flow conditions should not exceed 1.1 times the nominal pressure; during nominal-flow conditions, the pressure should not exceed 1.25 times the nominal pressure. The reseating pressure should be at least 0.8 times the nominal pressure.

The pressure–flow characteristics of safety valves are the relationship between the prevalve pressure and the flow rate of the safety valve. Once the safety valve’s preload, spring stiffness, and overlap are determined, variations in the safety valve’s flow rate induce corresponding changes in the valve port span. Furthermore, both the steady-state hydraulic pressure and the force exerted by the spring on the slide valve change, leading to variations in the prevalve pressure.

According to the dynamic balance of the safety valve, shown in Equation (3), when the roof pressure slowly changes, the roof is in an equilibrium state regarding the force acting on the main valve core. The upstream pressure of the safety valve is then calculated as follows:

(4) $$P=\frac{m(d^{2}X/dt)+R_{fa}(dX/dt)+K(X+X_{0})+F_{y}}{A_{0}}$$

**Figure 3:**
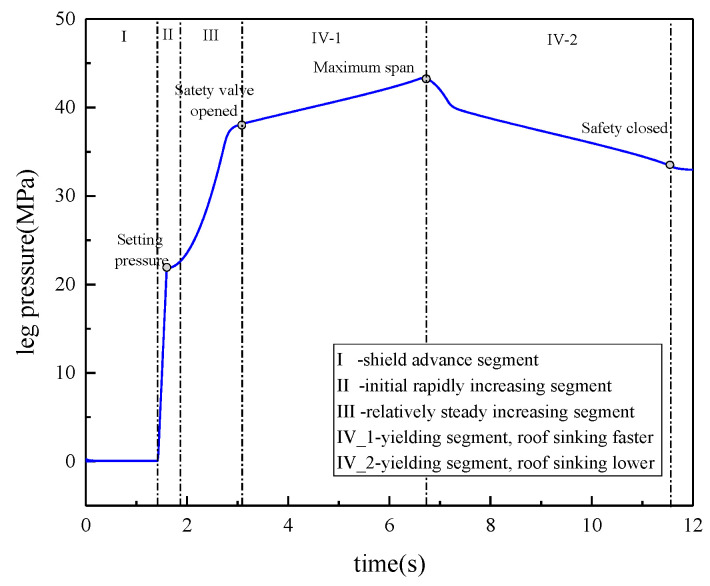
Pressure curve of the lower leg cavity.

In the simulation model shown in Figure 2, the column material is assumed to be rigid, and displacement loading is applied to the column. When the safety valve’s nominal flow rate is 320 L/min, the corresponding displacement loading velocity is 0.17 m/s. The input control modes for the control valve and roof loading are as follows:

(1) Control valve action: The control valve is in fluid feed mode, resulting in the column ascending, with the interaction with the roof lasting 1.6 s. The control valve remains in the neutral position for 10 s. (2) Roof displacement load: The initial gap between the roof and the column is 300 mm. Prior to roof catching, the roof’s speed remains at 0. Subsequent to roof catching, the roof’s motion splits into two stages, transitioning from 0 to 0.17 m/s and then from 0.17 to 0 m/s, each undergoing a 5 s simulation. Consequently, the duration of the total simulation is 11.6 s. The pressure in the lower chamber of the column is shown in Figure 3. The pressure curve can be divided into four segments according to the rate of pressure change. The first segment is the shield advance segment, where the pressure is maintained at approximately 0.05 MPa. The second segment is the initial rapidly increasing segment, where the leg rises, the hydraulic check valve closes, and the pressure is as high as 22 MPa. The third segment is a relatively steadily increasing segment, where the roof sinks, and the pressure slowly increases. The final segment is the yielding segment: the roof rapidly sinks, the safety valve opens, and the leg maintains constant resistance.

**Figure 4:**
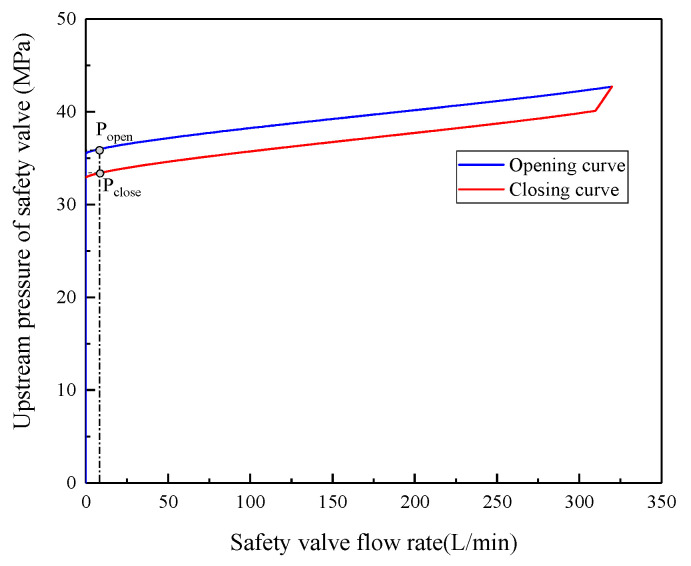
Static characteristic curve for fad320/40 safety valve.

The simulation results were processed to obtain the static characteristic curve (i.e., pressure–flow curve) of the safety valve, as shown in Figure 4. According to the simulation results, the opening pressure of the safety valve is 35.7 MPa, the return pressure is 33.1 MPa, the nominal pressure is 41.4 MPa, and the opening and closing pressure difference is 2.6 MPa. The safety valve opens and initiates overflow when the main valve port pressure reaches 35.7 MPa. The main valve port pressure reaches 41.4 MPa when the safety valve achieves the designed flow rate of 320 L/min. The spool experiences varying directions of frictional resistance during its opening and closing, leading to the closing pressure curve being lower than that of the opening pressure. The safety valve’s reseating pressure is 33.1 MPa, satisfying the design criteria.

#### 2.2.3. Analysis of Dynamic Characteristics of the Safety Valve

**Figure 5:**
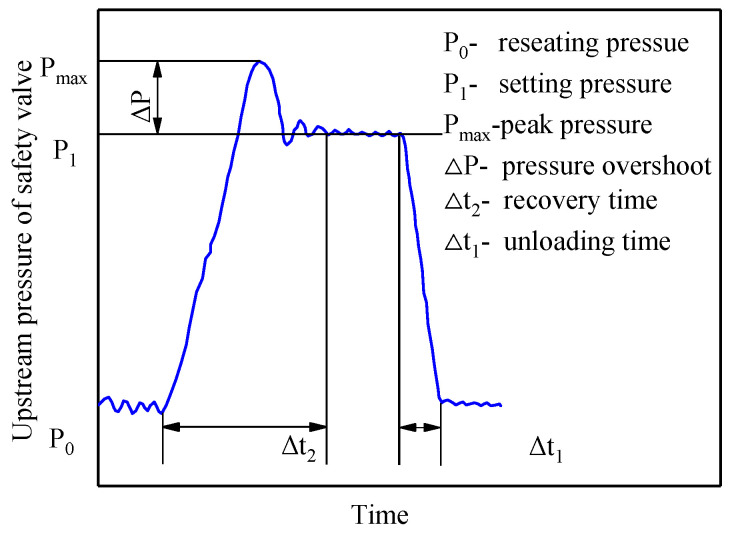
Dynamic characteristic curve of safety valve column.

Mining safety valves typically exhibit dynamic characteristics, characterized by abrupt shifts from a closed position (at pressure P_0_ to an open state and then returning to a closed state. During mining face retreat, the powered support beneath the roof experiences a sudden and intense load, causing an instantaneous and rapid increase in the hydraulic system pressure. During such instances, the safety valve must exhibit excellent dynamic characteristics to prevent damage to the hydraulic components or column cylinder explosions. The key indicators include the pressure overshoot (Δp), pressure recovery time ($\Delta t_{1}$), and unloading time ($\Delta t_{2}$), as illustrated in Figure 5.

**Figure 6:**
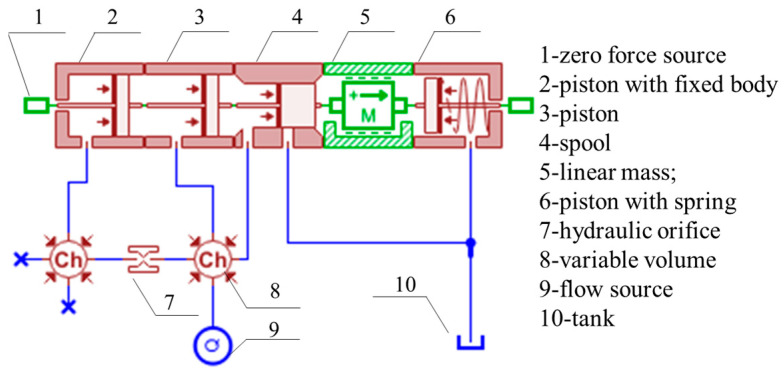
Simulation model of safety valve.

**Figure 7:**
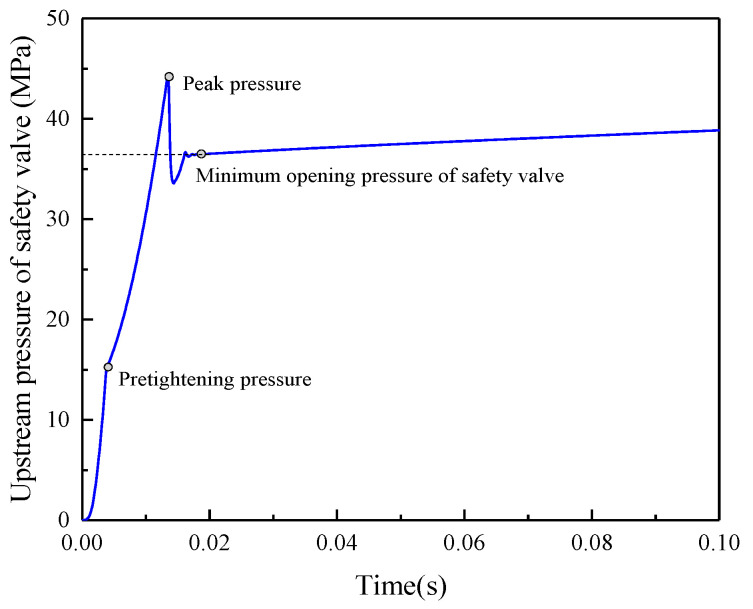
Dynamic characteristic curve of safety valve in AMESim.

The simulation model of the FAD320/40 safety valve was established according to the parameters in Table 1 (Figure 6). The simulation results in Figure 7 show an instantaneous pressure rise to 15.5 MPa (spring force of 3925 N) during the main valve’s opening phase and then a slow in the growth rate. The main valve experiences a pressure shock peak of 44.5 MPa at 13.5 ms and 36.5 MPa at 18.6 ms before stabilizing. The pressure overshoot is 8 MPa, which generally aligns with the values obtained from the field requirements.

### 2.3. Optimal Selection of Characteristic Parameters of Safety Valve

**Figure 8:**
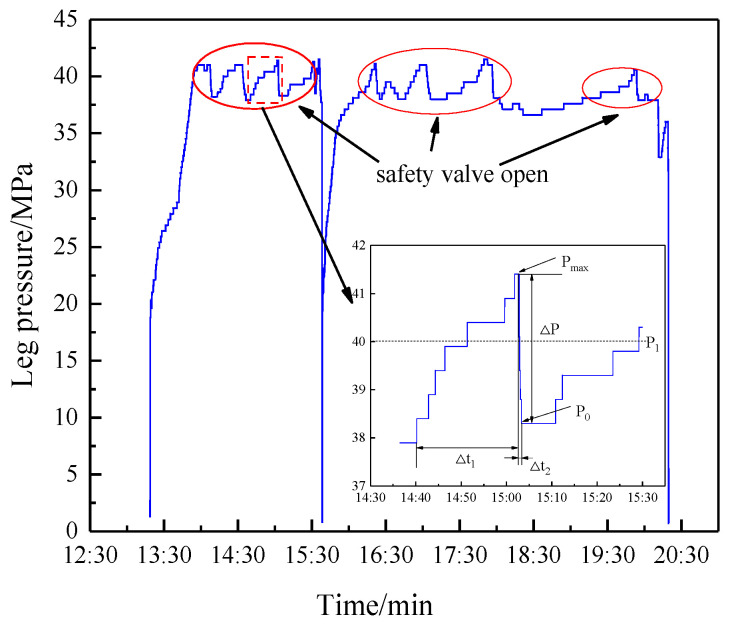
Reason for feature parameter extraction for safety valve.

In certain application scenarios, such as in nuclear power plants and oil exploration, where precise safety valve monitoring is crucial, the operational status is typically monitored by simultaneously tracking parameters such as the pressure, flow rate, and spool displacement [8,22]. In contrast, for cartridge safety valves employed in a powered support, where the monitoring precision is less critical, monitoring is primarily limited to the column pressure. The time–pressure curve of a typical safety valve opening is shown in Figure 8.

A GPD60 silicon piezoresistive pressure sensor (Zhengzhou Coal Mining Machinery Group Company Limited, Zhengzhou, Henan, China) was used for column pressure monitoring at the working face site, which has an accuracy of ±0.5% FS, a resolution of 0.1 MPa, and an acquisition frequency of 1 HZ. The characteristics of a safety valve under real working conditions are influenced by the hydraulic piping system, containers, other actuators, and the fluid–solid coupling interaction between the fluids and the slide valve, in addition to its structural parameters. By neglecting the influence of the working fluid medium and the hydrodynamic forces on the upstream pressure, the monitoring pressure could be approximated as the upstream pressure of the safety valve.

Based on the time–pressure curve of the powered support column, the characteristic parameters of the selected safety valve were determined, as shown in Figure 8, which included static characteristic indices such as the peak ($p_{\max}$), reseating ($p_{0}$), and blowdown (Δp) pressure, and dynamic characteristic indices such as the pressure recovery ($\Delta t_{1}$) and unloading ($\Delta t_{2}$) time. The specific definitions of these indices are as follows:

Peak pressure ($p_{\max}$): This refers to the pressure at the valve’s inlet when the flow through the safety valve reaches its maximum. Definition: The peak pressure represents the highest point on the pressure curve as the valve transitions from a closed state to an open state and then back to a closed state.

Reseating pressure ($p_{0}$): This refers to the pressure at which the safety valve spool is about to close while allowing only 1% of the rated flow rate. Definition: The reseating pressure represents the minimum point of the pressure curve as the valve transitions from opening to closing states.

Blowdown pressure (Δp): This is the difference between the peak pressure and the reseating pressure.

Recovery time ($\Delta t_{1}$): This refers to the duration required for the pressure to increase from the reseating pressure to its peak value.

Unloading time ($\Delta t_{2}$): This refers to the duration required for the pressure to reduce from the peak pressure to the reseat pressure.

## 3. Method of Extracting Characteristic Safety Valve Parameters Based on Extreme Value Point

The roof pressure, in the process of safety valve opening and closing, is characterized by a notable fluctuation. The extreme point of these pressure fluctuations is related to the limit position of the safety valve spool displacement and is strongly correlated to the characteristic safety valve parameters, so the extreme point extraction algorithm can be used to extract the characteristic safety valve parameters based on the roof pressure data.

**Figure 9:**
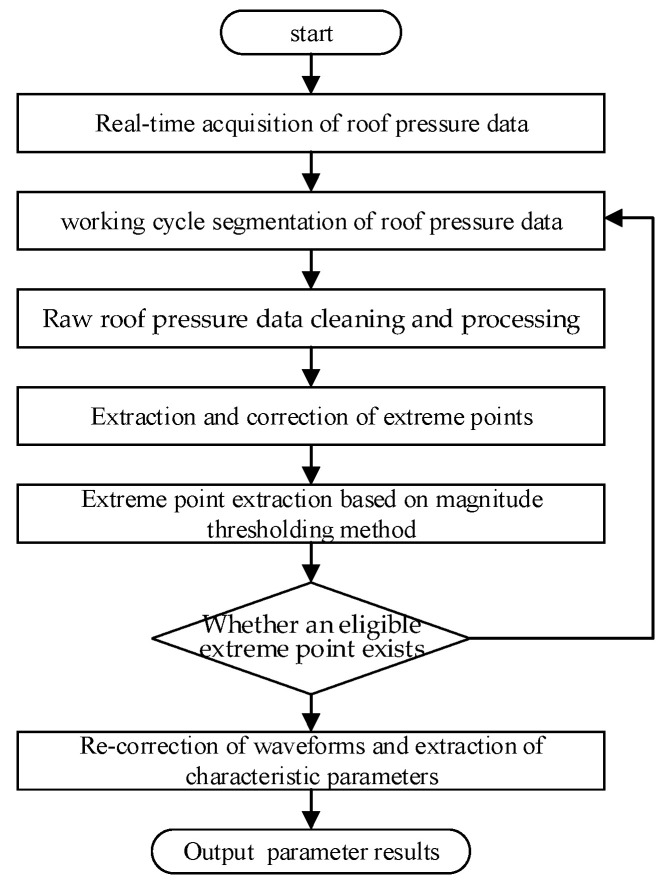
Flow chart of method of extracting characteristic safety valve parameters.

A flow chart of the extraction method is shown in Figure 9. From the panel, real-time roof pressure data can be obtained through the mine’s pressure monitoring system. First, the roof pressure data were segmented according to the information on the lowering and raising of the supports, and the roof pressure data from the last working cycle were selected as the study objects. Second, the raw roof pressure data were cleaned and preprocessed through a data smoothing process. Third, the comparison discriminant method was used to identify the extreme value points, and the beginning and end points of the extreme values were corrected. Fourth, the extreme value points were filtered using the amplitude threshold method. Fifth, if the extreme value points satisfied the conditions, the waveforms were recorrected, and the characteristic parameters were extracted. Finally, the results of the characteristic safety valve parameters were output.

### 3.1. Working Cycle Segmentation of Roof Pressure Data

Zhao Jiyu [23] introduced a sliding window approach to identify the support’s working cycles. Wang Liuwang et al. [24] proposed a novel method based on an adaptive dual threshold to extract the fundamental parameters of partial discharge. When the support controller operates the support, it undergoes three sequential steps: lower, advance, and raise. In this study, we identified the pressure just before the support-lowering signal as the end point for the previous working cycle, with the pressure after the support-raising signal as the start point for the current working cycle based on a linkage analysis of the electrohydraulic control system’s manipulation signals. Upon determining the end of the previous work cycle, the analysis within that cycle commenced.

### 3.2. Roof Raw Pressure Data Cleaning and Preprocessing

The raw pressure data were multiplatform-type data, which interfered with the peak and trough identification. A discriminant method was employed to initially filter the extreme value points using a comparative approach. When the condition $P_{i-1}>P_{i}\leq P_{i+1}$ was met, minimum points were identified, and when the condition $P_{i-1}<P_{i}\geq P_{i+1}$ was met, maximum points were identified. In Equation (5), matrix X represents the set of raw data points that resulted in a smoothed dataset denoted as Y1n×2. The finally obtained smooth extreme value envelope is illustrated in Figure 9a.

(5) $$\left\{\begin{array}{l} X={\left[\begin{array}{l} T_{1},T_{2},\ldots ,T_{i},\ldots ,T_{m}\\ P_{1},P_{2},\ldots P_{i},\ldots ,P_{m} \end{array}\right]}^{T}\\ i=1,\ldots ,m \end{array}\right\}\Rightarrow \left\{\begin{array}{l} Y={\left[\begin{array}{l} T_{1},T_{2},\ldots ,T_{j},\ldots ,T_{n}\\ P_{1},P_{2},\ldots P_{j},\ldots ,P_{n} \end{array}\right]}^{T}\\ j=1,\ldots ,n \end{array}\right\}$$

where X is the data from the pressure monitoring system, T is the time of the pressure data, P is the pressure value, i is the amount of data, Y is the smoothed data, and j is the amount of smoothed data.

### 3.3. Extraction and Correction of Extreme Points

(1) A discriminant method was employed to filter the extreme value points through a comparative approach. In Y when the condition $P_{j-1}>P_{j}<P_{j+1}$ was met, the minimum points were identified, and when the condition $P_{j-1}<P_{j}>P_{j+1}$ was met, the maximum points were identified.
(2) The beginning and end points of the extreme values were corrected. After the extraction of the extreme value points, the starting and ending points of the dataset were both considered maximum points. According to the reason for the extraction of the feature parameters of the safety valves (Figure 8), the dataset was extended at both the beginning and end of the cycle to calculate the pressure recovery ($\Delta t_{1}$) and unloading ($\Delta t_{2}$) times.

The initial value at the beginning of the pole was defined as the nearest point to the reseating pressure between the starting point of the working cycle and the first maximum point. The initial value at the ending of the pole was defined as the nearest point to the reseating pressure between the end point of the working cycle and the last maximum point, with the corrected dataset denoted as z.

### 3.4. Extreme Point Extraction Based on Magnitude Thresholding Method

(1) Peak wave identification: By conducting a statistical analysis of the prevalve pressure signal of the safety valve, we observed that the peak wave associated with the safety valve’s opening fluctuates within a defined range. The fluctuation range before and after the safety valve’s opening exceeds 0.5 MPa but remains below 0.2 times the regulated pressure. The longitudinal threshold ($P_{\mathrm{pm}}’$) was calculated as the smaller value between the average peak pressure of the previous three occurrences minus twice the mean square deviation and the theoretical value. As the time interval (Δt) between the successive safety valve openings exceeds 1 min, a neighborhood time is set to 1 min. The condition for determining the actual wave peak point ($Z[j_{pm}]$) is as follows:
  (6) $$\left\{\begin{array}{l} P_{pm}{}^{\prime }=\min\{P_{theory},\bar{P_{pm}}-\sigma_{pm}\}\\ Z[j_{pm}]\}\geq P_{pm}{}^{\prime }\\ Z[j_{pm}]\geq Z[j],j\subseteq [t-\Delta t,t+\Delta t] \end{array}\right\}$$
  where $\Delta =P_{1}\times 0.2$ is the set threshold value, Δt is initially set to 1 min as the neighborhood time, and $j_{pm}$ is the position of the peak point.

(2) Safety valve trough identification: The trough value was further divided into left and right trough values for assessment.

(7) $$\left\{\begin{array}{l} \max\{Z[j]-Z[j_{pm}]\}\leq \Delta \\ \min\{Z[j]-Z[j_{pm}]\}\geq \Delta p\\ Z[j_{pm},0]\leq Z[j_{pl},0]\leq Z[j_{p(m+1)},0]\\ Z[j_{pl},1]\geq Z[j,1],j\subseteq [Z[j_{pl},0]-\Delta t,Z[j_{pl},0]+\Delta t] \end{array}\right\}$$

The results of the extreme point identification are shown in Figure 9b.

### 3.5. Recorrection of Waveforms and Extraction of Characteristic Parameters

**Figure 10:**
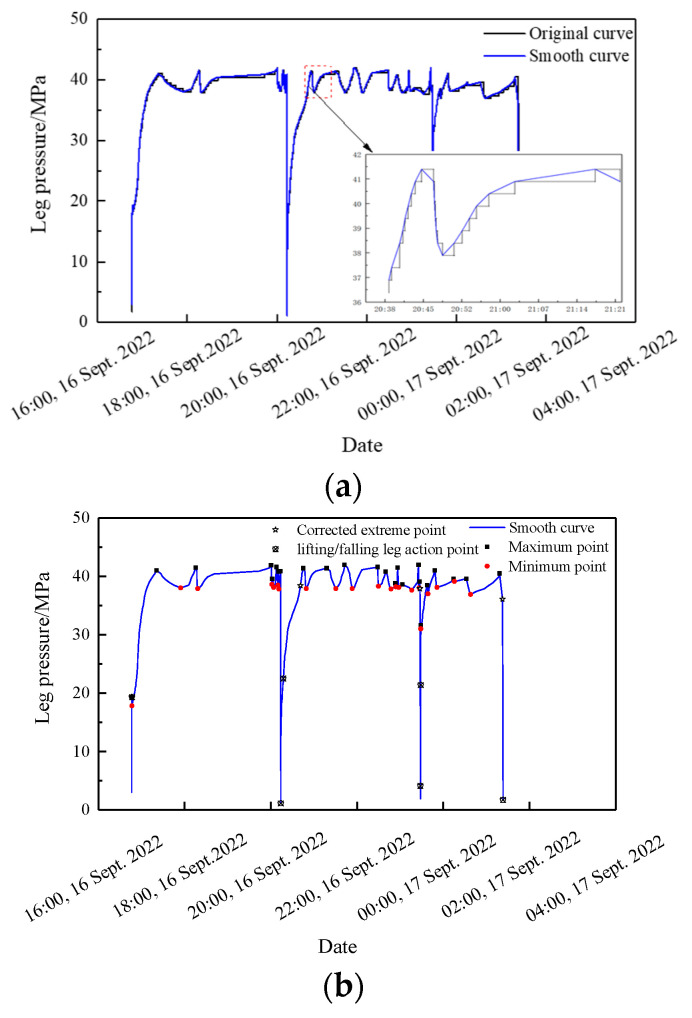

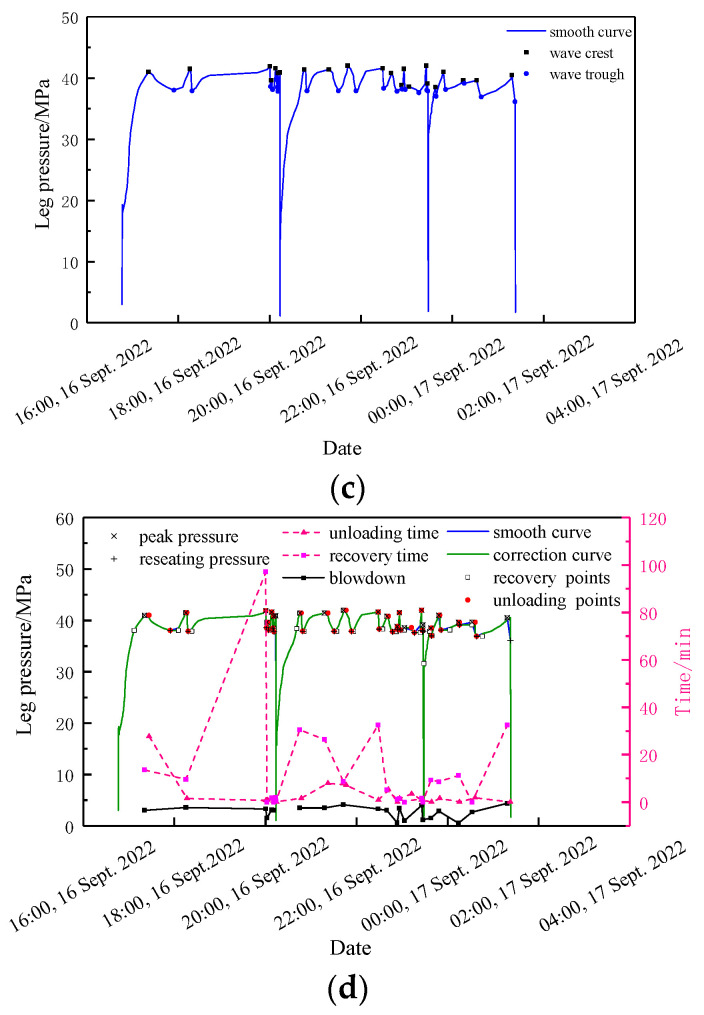
Extraction of pressure signal for safety valve: (**a**) roof raw pressure data cleaning and preprocessing; (**b**) extraction and correction of extreme points; (**c**) extreme point extraction based on magnitude thresholding method; (**d**) recorrection of waveforms and extraction of characteristic parameters.

Employing the extreme value envelope, as described in Section 3.2, effectively removed the plateau value located to the right of the extreme point. When the safety valve smoothly opens, the prevalve pressure is intentionally maintained in close proximity to both the peak and trough for a specific duration, as depicted in Figure 4. By referencing the positions of the peak and trough points, a reverse search of the raw pressure data is conducted to ascertain the optimal time to maintain proximity to the peak/trough, resulting in the final curve shape illustrated in Figure 10d.

## 4. Ground Pressure Analysis and Safety Valve Condition Evaluation Based on Characteristic Parameters

### 4.1. Ground Pressure Analysis

#### 4.1.1. Overburden Structure Analysis at Panel 0116^3^06 with Top Coal Caving

**Figure 11:**
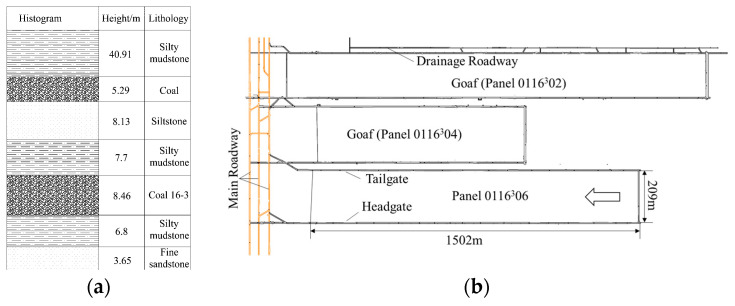
Geological and mining conditions: (**a**) partial stratigraphic sequence; (**b**) layout of panel 0116^3^06.

We took panel 0116^3^06, which was experiencing top coal caving in eastern Inner Mongolia, as an example. This panel was 209 m wide and 1502 m long with a 20 m wide barrier pillar between the panels; partial stratigraphic sequence is shown in Figure 11a, and the layout is shown in Figure 11b. The main coal seam was −218m deep and −8.46m thick, and dipped 3° on average. The mining height was 2.7 m, the mining to top coal caving height ratio was 1:2.1, and the single-wheel sequential coal release mining process was applied.

The panel was equipped with 140 ZF9000/17/32 supports and a network-type electro-hydraulic control system, to which built-in pressure sensors were mounted on each column to monitor the pressure in both the front and rear columns.

**Figure 12:**
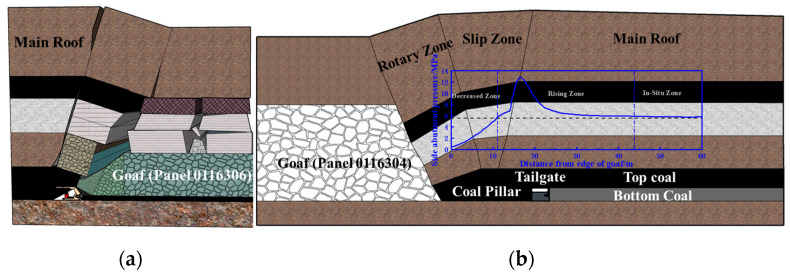
Rock structure characteristics of panel 0116^3^06: (**a**) inclination and (**b**) strike of panel.

Compared with the ordinary comprehensive mining working face, the mining height of this panel was 8.4 m and was characterized by a substantial overburden rock collapse space. Here, the thickness and layers of the immediate roof increased, and the main roof moved upward [25]. The immediate roof consisted of top coal, silty mudstone, siltstone, and an upper coal seam. Notably, the upper coal seam was left unmined because of its higher gangue content. The main roof was 40 m thick massive silty mudstone. During the mining process, the overburden collapsed in a medium-thick laminated form, and a masonry beam structure formed at the main roof, which rotated or slipped, resulting in a roof weighting, as shown in Figure 12a.

According to the test results of the physical and mechanical parameters of panel 0116^3^06, the uniaxial compressive strength, tensile strength, and elasticity modulus of the main roof’s silty mudstone were 13.18 MPa, 1.23 MPa, and 1.52 GPa, respectively. The results of the key strata theory [26] analyses indicated that the main roof load was 985.04 kPa, and the fracture distance was 10.78 m.

The goaf was adjacent to the outside of the tailgate, with a 20 m wide barrier pillar. Because the overburden structure in the deep coal body was broken, the key blocks rotated, generating friction, extrusion pressure, and moment interactions with the adjacent rock layers. Ultimately, a substantial bending moment force was generated between the triangular slip zone and the unfractured rock layer, intensifying the ground pressure behavior, as shown in Figure 12b.

According to the theory of delta equilibrium [27], the lateral support stress distribution was divided into three zones: decreasing (0~10.4 m), rising (10.4~44.2 m), and in situ (>43.8 m) zones. A stress peak formed at 16.2 m, with a peak size of 12.2 MPa, where the coal cohesion, elastic modulus of the interlayer rock strata, elastic modulus of the key strata, mining height, support resistance of the coal pillar side, and the stress concentration coefficient were 1.8 MPa, 2 GPa, 6 GPa, 8 m, 0.5 MPa, and 2, respectively, as shown in Figure 12b.

#### 4.1.2. Ground Pressure Analysis Based on Characteristic Parameters

The method of extracting characteristic parameters, described in Section 3, was utilized to analyze the roof pressure data from 140 supports in panel 0116^3^06 in September 2022. The relationship between the safety valve opening times and the ground pressure was analyzed from the perspective of panel inclination and strike.

#### Analysis of Inclined Ground Pressure Based on Safety Valve Opening Times

The frequency of the safety valve openings on all supports in the panel reflects the characteristics of the inclined ground pressure. The four-leg support was equipped with two safety valves separately monitoring a single leg’s pressure in the front and rear rows.

**Figure 13:**
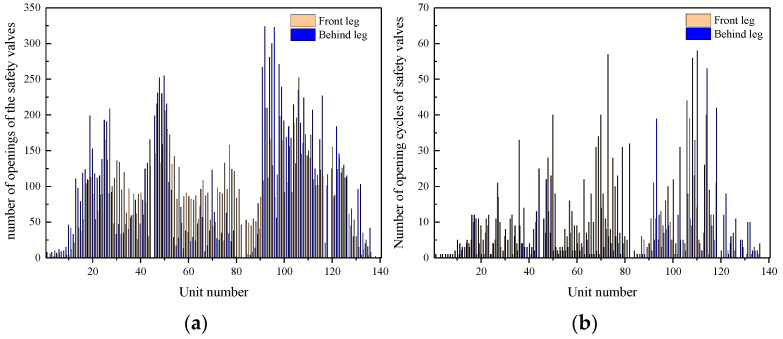
Statistics of safety valve opening frequency: (**a**) number of openings; (**b**) number of opening cycles.

Determination of safety valve opening times: The safety valve opened following the process of starting the overflow, maximum opening, and then returning to seat. Correspondingly, the recovery point, peak pressure, and reseating pressure process formed in the characteristic parameter extraction results. Therefore, the safety valve opening time was defined as being equal to the amount of peak pressure. The statistics of the safety valve opening times of the front and rear columns are shown in Figure 13a.

Determination of safety valve opening cycle times: Within each working cycle, the safety valve may remain closed, open once, or open frequently. If the safety valve opened once or opened frequently during each working cycle, this was considered an instance of safety valve opening. The statistics of the safety valve open cycle times are shown in Figure 13b.

By comparing the on-site mining pressure and theoretical model, the statistics of the safety valve opening frequency were found to reflect the distribution characteristics of the ground pressure in the inclined panel. The specific analysis was as follows:

(1) Reference [28] demonstrated that the leg pressure in super long panels exhibits a distribution characterized by a saddle- or W-shaped pattern. The number of openings of the safety valves also follows a W-shaped distribution, with peak values observed at supports 44 and 92. Similarly, the number of opening cycles of the safety valves exhibit a W-shaped distribution, with peak values found at supports 32, 58, and 92. Considering the regulation of on-site ground pressure, the analysis of the number of safety valve opening cycles provides enhanced accuracy and clarity.
(2) The number of safety valve opening cycles for the headgate was substantially higher than at other locations. The headgate was in proximity to the goaf, where the lateral support pressure from the adjacent goaf had a strong influence, resulting in intense ground pressure. The number of safety valve opening cycles in supports 1 to 38 noticeably increased, which is in agreement with the theoretical calculation of a pressure rise in the 50 m pressure increase zone, as described in Section 4.1.2.
(3) The opening frequency of the front column safety valves was noticeably higher than that of the rear column in the middle panel (supports 50 to 90). In the middle panel, the top coal was prone to caving, leading to the frequent occurrence of goaf formations in the rear column. As a result, the pressure transferred to the front column during mining, causing a higher opening frequency of the safety valves in the front compared with those in the rear. On average, the safety valves opened and closed 10.4 times per work cycle, indicating frequent openings during roof weighting and suggesting the presence of high-intensity ground pressure.

##### Analysis of Strike Ground Pressure Based on Safety Valve Opening Ratio

The safety valve opening ratio is a core indicator used in ground pressure monitoring in the panel. Cheng Jingyi [29] developed a support and roof state intelligent (SSRI) sensing system for a fully mechanized mining face and introduced an intelligent method for analyzing the ground pressure. This paper proposes a method to determine the safety valve opening ratio based on this system.

First, automatically search for the starting and ending times of roof weighting in the SSRI system. Second, count the number of supports with safety valve openings during the entire duration of roof weighting (without double counting). Lastly, calculate the safety valve opening rate by dividing the number of supports with the safety valve openings by the total number of supports on the panel.

The corresponding results are presented in Table 2. Four roof weightings occurred in September. The average distance, duration, pressure intensity, dynamic loading coefficient, safety valve opening ratio, and increased resistance were 15.75 cycles (10.5 m), 1.5 cycles (1.05 m), 25.70 MPa, 1.25, 0.05, and 5.25 MPa, respectively.

### 4.2. Analysis and Evaluation of Safety Valve Condition

#### 4.2.1. Analysis and Evaluation of Condition of Single Safety Valves

**Figure 14:**
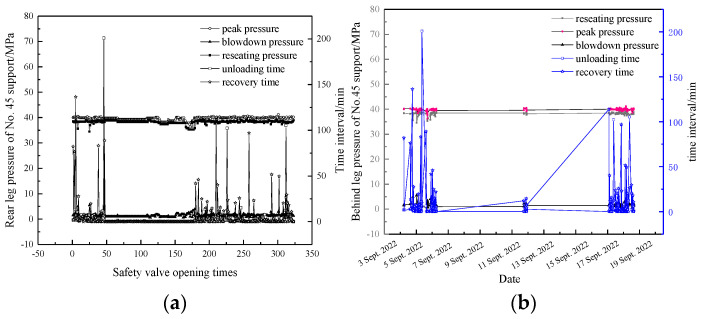
Inter-relationships among the characteristic parameters of a single safety valve: relationship between the characteristic parameters and (**a**) safety valve openings and (**b**) time.

The rear leg pressure of the no. 45 support in the panel, as an example, was separately analyzed from the perspectives of the safety valve opening times and duration, with the results shown in Figure 14.

(1) The service life and failure probability of the safety valve are strongly related to the number of valve openings [30]. The FAD320/40 safety valve, examined in this study, has a recommended cycle count of 3000 times, as specified by the manufacturer. The peak pressure typically reaches around 40 MPa, with the reseating pressure being approximately 1.3 MPa lower. The unloading and pressure recovery times generally range from 0 to 5 min. As the number of valve openings increases, the various parameters of the safety valve generally remain within an acceptable range. Based on the safety valve’s opening and closing characteristics (Figure 4), a maximum opening pressure of 45.8 MPa corresponds to a flow rate of 330.2 L/min, which essentially satisfies the on-site application requirements.
(2) The temporal and spatial characteristics of safety valve opening indicate some of the main forms of strata movement in the panel [12]. In the panel with top coal caving, the evolution of the ground pressure exhibits distinct stages of strata fracturing and instability [31]. During the strata fracturing stage, the roof slowly subsides, and the safety valve partially opens. In the instability stage, the safety valve experiences frequent opening and closing cycles. The distribution of pressure recovery and unloading durations of the safety valve indicates its ability to adapt to the slow and rapid subsidence of the roof in this working environment.

#### 4.2.2. Condition Analysis and Evaluation of all Safety Valves

**Figure 15:**
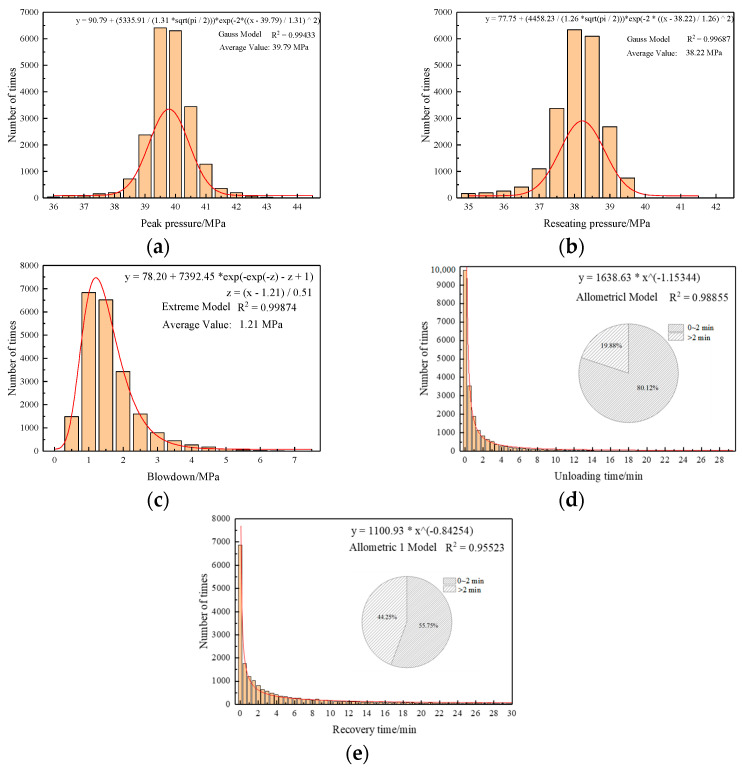
Overall distribution status of characteristic parameters for safety valves: (**a**) peak pressure, (**b**) reseating pressure, (**c**) blowdown pressure, (**d**) pressure recovery time, and (**e**) unloading time.

The characteristics of all 140 supports in the panel, including 280 online monitoring safety valves, were analyzed using macro statistics. The peak, reseating, and blowdown pressures demonstrated overall Gaussian distributions, with average values of 39.79 MPa, 38.22 MPa, and 1.21 MPa, respectively, as shown in Figure 15a–c, satisfying the specifications provided by the safety valve’s manufacturer.

The numbers of pressure recoveries and unloadings of the safety valve followed exponential distributions, predominantly concentrating within the 0 to 2 min range, indicating the valve’s high sensitivity. The distribution of the unloading duration primarily depended on the valve’s structure, with approximately 80.12% of unloading occurring within the 0 to 2 min range, facilitating rapid unloading, as shown in Figure 15d. The pressure recovery duration was influenced by both the roof subsidence and the valve’s structure, with approximately 55.75% of the pressure recovery time falling within the 0 to 2 min range, which is consistent with the actual on-site conditions, as shown in Figure 15e.

### 4.3. Development and Application of Safety Valve Status Monitoring Module

To enable the precise online monitoring of the statuses of the safety valves and acquire operational parameters during application, a standardized database must be established for safety valve fault diagnosis to provide essential data and analyze the pressure conditions in a panel. The safety valve status monitoring module comprises five submodules: parameter configuration, a comprehensive overview of the safety valve status, statistics on the safety valve opening ratio, individual querying and maintenance, and report statistics.

#### 4.3.1. Parameter Configuration

A comprehensive parameter table used to configure safety valve models was developed. By default, the model remains consistent throughout the whole panel; if the parameters vary due to the replacement of certain safety valves in a single support, the safety valves can be individually set. The safety valve’s basic parameters include simple, standard, and complex modes, which can be configured based on specific requirements.

#### 4.3.2. Comprehensive Overview of Safety Valve Status

**Figure 16:**
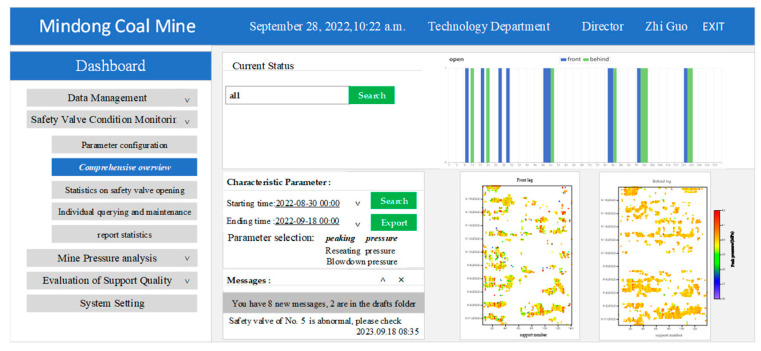
The overall display of safety valve status.

Through an automatic extraction and analysis of the five characteristic parameters by the system, the overall list of safety valve statuses was formed with the X coordinate as the support number, the Y coordinate as the time, and the Z coordinate as the characteristic parameter to provide a macroscopic overview of the overall safety valve opening status on the time scale. Furthermore, the data linkage function enables engineers to access and display comprehensive pressure data within a specific working cycle by clicking on the corresponding data point in the table. The actual dashboard is displayed below in Figure 16.

#### 4.3.3. Safety Valve Opening Statistics

**Figure 17:**
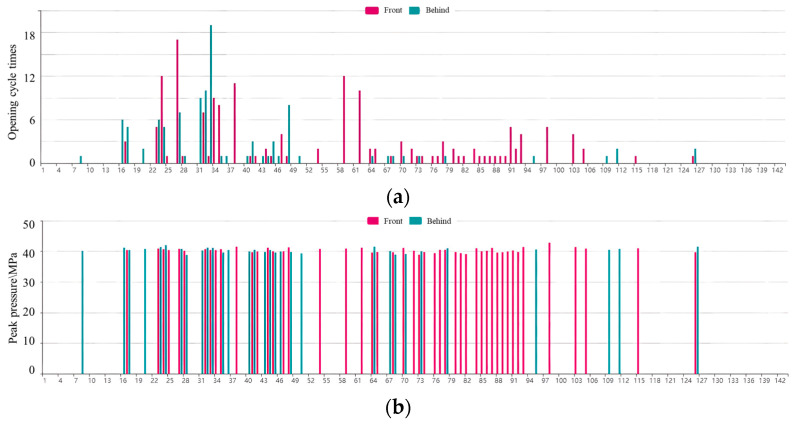
Statistics of safety valve opening rate: (**a**) number of safety valve opening cycles; (**b**) peak pressure.

The distribution of the ground pressure in a panel can be reflected using the number of safety valve opening cycles on inclined supports, as shown in Figure 17a. Moreover, a larger ground pressure in the panel and a higher overflow flow rate of the safety valves result in an increased peak pressure. Further statistical details on the peak pressure are shown in Figure 17b.

#### 4.3.4. Individual Querying and Maintenance

The five parameters described in this article can be queried, including the scales for time and the number of safety valve openings, and the characteristics of their distribution can be presented. The relationship between the opening pressure, reseating pressure, blowdown pressure, and the number of safety valve openings can be used to clarify the states of the safety valves as the number of cycles changes. Moreover, based on the classical bathtub curve lifespan prediction model and the trend-based fault diagnosis model, the lifespan of safety valves can be predicted, and their faults can be diagnosed online.

Regarding safety valve maintenance, during the on-site inspection and maintenance process, the identified faults and types of safety valves should be recorded and managed in a ledger, which provides data samples for subsequent adaptive and precise online fault diagnosis using machine learning methods.

#### 4.3.5. Report Statistics

This module is primarily used to generate reports that consolidate the basic equipment information of the safety valves, the statistical data on their opening conditions, the distribution patterns of various characteristic parameters, and prediagnosis information on faults. These reports facilitate the precise management of powered support safety valves during mining operations.

## 5. Conclusions

(1) A mathematical model and a simulation model called AMESim were established to precisely determine the static and dynamic characteristics of the FAD320/40 safety valve. The minimum opening pressure of the safety valve was 35.7 MPa, and the main valve’s orifice pressure corresponding to the nominal flow rate was 41.4 MPa, with a pressure overshoot of 8 MPa.

(2) A method of extracting the characteristic safety valve parameters based on extremum point detection was developed. By considering the on-site roof pressure monitoring, the principles and methods for extracting five characteristic parameters (static characteristics: peak, reseating, and blowdown pressure; dynamic characteristics: pressure recovery and unloading times) were determined.

(3) The relationship between the safety valve openings and the on-site mine pressure was analyzed, and the state distribution of the characteristic safety valve parameters was clarified. The results indicated that this method enables the accurate online statistical analysis of safety valve openings.

(4) Building upon the SSRI system, a safety valve state monitoring module was developed, offering functions such as parameter configuration, a comprehensive overview of the safety valve’s status, statistics on the safety valve’s opening ratio, individual querying and maintenance, and report statistics. This module was practically applied in mines.

## Figures and Tables

**Table 1:** Model validation parameters.

	Name	Element	Parameter	Value
1	emulsion	FP04	density (kg/m³), bulk modulus (MPa), absolute viscosity (cP)	998, 1950, 51
2	pump		flow rate (L/min)	400
3	unloading valve	RV012	relief valve cracking pressure (bar)	315
4	column	BAP12	piston diameter (mm), rod diameter (mm)	200, 0
		BAF01	external piston diameter (mm)	225
			clearance on diameter (mm), contact length (mm)	0.01, 300
		BAP12	piston diameter (mm), rod diameter (mm)	200, 185
		MECMAS21	mass (kg), inclination (degre), coefficient of viscous friction (N/(m/s))	121, −90, 80
			higher displacement limit (m), lower displacement limit (m)	2.8, 1.7
		LSTP00A	gap or clearance with both displacements zero (mm)	300
5	control valve	BAO012	spool diameter (mm), rod diameter (mm),	20, 10
			underlap corresponding to zero displacement (mm)	−2
		BAO011	spool diameter (mm), rod diameter (mm)	20, 10
6	hydraulic check valve	BAP12	piston diameter (mm), rod diameter (mm)	20, 0
		MECMAS21	mass (g), coefficient of viscous friction (N/(m/s))	120, 500
			higher displacement limit (mm), lower displacement limit (mm)	5, 0
		BAP016	piston diameter (mm), rod diameter (mm),	31, 12
			spring force at zero displacement (N), spring rate (N/mm)	80, 7
		LSTP00A	gap or clearance with both displacements zero (mm),	1
		BAP026	diameter of poppet, poppet half angle	17.5, 60
			diameter of hole, diameter of rod (seat side)	13, 0
		MECMAS21	mass (g), coefficient of viscous friction (N/(m/s))	40, 100
			higher displacement limit (mm), lower displacement limit (mm)	5, 0
		BAP016	piston diameter (mm), rod diameter (mm)	22.5, 17
			spring force at zero displacement (N), spring rate (N/mm)	45, 15
7	safety valve	BAP12	piston diameter (mm), rod diameter (mm)	13, 0
		BAP12	piston diameter (mm), rod diameter (mm)	13, 0
		BAO042	number of orifice holes, spool diameter (mm), rod diameter (mm)	10, 8, 0
			hole diameter (mm), underlap corresponding to zero displacement (mm)	2, −6
		MECMAS21	mass (g), stiction force (N), Coulomb friction force (N)	80, 400, 400,
			coefficient of viscous friction (N/(m/s))	1000
			higher displacement limit (mm), lower displacement limit (mm)	8, 0
		BAP016	piston diameter (mm), rod diameter (mm)	42, 6
			spring force at zero displacement (N), spring rate (N/mm)	3925, 1144

**Table 2:** Roof weighting statistics.

No.	Advance/Cycle	Start Time	Duration/Cycle	End Time	Distance/Cycle	Pressure Intensity/MPa	Valve Opening Ratio	Increased Resistance/MPa	Dynamic Loading Coefficient
1	137	09–04 17:52	3	09–05 06:55	15	27.16	0.06	7.37	1.32
2	154	09–12 16:31	1	09–12 19:49	16	24.77	0.02	4.74	1.22
3	172	09–19 12:51	2	09–20 22:17	17	25.95	0.07	4.76	1.27
4	188	09–28 12:03	2	09–28 20:22	15	24.93	0.05	4.13	1.19
avg	-	-	1.5	-	15.75	25.70	0.05	5.25	1.25

## Data Availability

Some or all data, models, or code generated or used during the study are proprietary or confidential in nature and may only be provided with restrictions.
